# Adult vaccination in India: status and the way forward

**DOI:** 10.1080/21645515.2019.1692564

**Published:** 2019-12-06

**Authors:** Chandrakant Lahariya, Pankaj Bhardwaj

**Affiliations:** a Department of Health Systems Development, World Health Organization Country Office for India, New Delhi, India; b Department of Community & Family Medicine, All India Institute of Medical Sciences (AIIMS), Jodhpur, India

**Keywords:** Vaccines, India, expanded program of immunization, adult vaccination, Universal Immunization Program

## Abstract

In the last two decades, the childhood vaccination coverage in most low and middle-income countries including India has increased. Additional vaccines are being offered through national immunization programs as well as through private sector and the benefits of vaccination are reaching to more children than ever. This has resulted in major decrease in vaccine preventable diseases and contributed to decline in the morbidity and mortality rates. This development is expected to result in epidemiological transition (which is already happening) and mandates for policies and strategies to extend the benefit of available vaccines and vaccination beyond traditionally target age groups to include the adults, elderly and the at-risk populations. This article reviews the present status of adult vaccination in India and proposes a few approaches to move towards life course vaccination.

Vaccination is amongst the most cost effective public health interventions. The importance has been further highlighted by global eradication of small pox virus and disease and the near elimination of wild polio virus through vaccination. The World Health Organization (WHO) launched Expanded Program of Immunization (EPI) in 1974,^1^ and most countries followed with the launch of their National Immunization Programs (NIPs).^2^ India launched EPI in 1978, which was rechristened as Universal Immunization Program (UIP) in 1985.^2^ Under the UIP in India, the initial target was to achieve universal coverage, with the vaccines offered, by 1990; however, for long, the immunization coverage in India remained relatively low with wide inter-state variations.^2^ These amongst others were the reasons given that India did not add any new antigen in UIP till early 2007. During the same period, a number of new vaccines had been licensed, available, recommended for and included in NIPs of many countries.^3^

Starting year 2008 till mid of year 2019, a series of interventions in India, both at the national and sub-national level, resulted in improvement in immunization coverage and the antigens offered through UIP have nearly doubled.^4-6^ This became possible due to improved vaccine preventable disease (VPD) surveillance & adverse events following immunization (AEFI) reporting system; strengthened cold chain systems, capacity building of various groups of health staff; release of national vaccine policy, strengthening institutional mechanisms (National technical advisory group on immunization or NTAGI & setting up of immunization technical support unit (ITSU); attention on immunization system strengthening and implementing communication strategies focused on improved vaccine acceptance, amongst other.^6-8^ The foundation for new vaccines introduction was arguably established with introduction of Hepatitis B vaccine and *Haemophilus influenzae* type b (Hib) vaccines in UIP between 2008 and 2012 and by conducting catch up campaign for measles containing vaccines second dose (MCV2) staring in 2010.^2,9,10^ In the years to follow, initiatives such as Intensified Routine Immunization (IRI); Mission Indradhanush (MI) and Intensified Mission Indradhanush (IMI) contributed to immunization systems strengthening in India.^6-8^ In year 2019, there are 11 antigens as part of UIP and full immunization (FI) coverage has reportedly crossed higher than ever in the history of UIP in India (personal communication with National Program Manager).

The UIP in India, as with most other low and middle-income countries (LMICs), had been started with aim to achieve universal coverage of infants and children as well as pregnant women with vaccines offered. Tetanus toxoid (TT) vaccination for pregnant women was only adult component at the beginning. Later, Japanese encephalitis (JE) vaccination for adult population was included as part of the program in endemic districts.^2,11^ Catch up campaigns for measles second dose and rubella containing vaccines brought the adolescent population, in ambit of government vaccination efforts. Now, with Rubella vaccination being part of UIP and human papilloma virus (HPV) vaccine being considered for possible inclusion, adolescent vaccination is expected to become integral to UIP.

More than four decades after EPI and UIP, the vaccination programs globally and in India have matured. Coverage is higher, and more vaccines are available and recommended. The programs are continuously learnings from changing epidemiology. The increased and sustained vaccination coverage has results in epidemiological shift with the disease prevalence in higher age group. The traditional childhood vaccination programs world over are transitioning toward ‘child and adolescent vaccination programs’. The vaccination of adult is a natural next step to maximize the full benefits of the proven and lifesaving interventions that are vaccines.

It is the time, for all countries to closely examine issue of offering vaccines to additional populations, i.e., adult population.

This issue of human vaccines and immunotherapeutic (HVI) has published a narrative review highlighting the burden of VPDs in India and argues for initiatives for stepping up adult vaccination.^12^ The steps suggested include effective health education for public to understand the risks of VPDs and the benefits of vaccination. At the same time, ensuring that the health care system has an adequate capacity and strategies to deliver vaccines to adults as well as adequate financing mechanisms to support the expanded delivery of vaccines to adults. In fact, adult vaccination is an issue, which most LMICs need to examine and take informed decision.

Traditionally NIPs in many LMICs, have focused on vaccination for infants and children. In last three decades, additional vaccines have become available vaccines for adults and high-risk groups and are being used sub-optimally, in most countries and not a phenomenon restricted to LMICs. However, in epidemic prone areas, a few vaccines are offered to mass and the countries have adopted that approach as well.^13^ There are a number of vaccines at various stages, benefits of which, can be optimized if adult population is also offered and systematically vaccinated. In context of the article published in this issue of journal,^12^ it can be noted that adult vaccination has not often been considered as a major technical discussion including that it was not discussed in the first and only national vaccine policy of India in 2011.^14^ Adult vaccination, a few times, has been deliberated in meetings of National Technical Advisory Group on Immunization in India, yet NTAGI does not have clear mandate to provide recommendations on vaccination for adults, beyond UIP.^15^

In last decade, vaccination of health workers as high-risk group for hepatitis B vaccine etc. has resulted in a few large institutions offering vaccination for their employees. However, health being a state subject in federal systems of governance in India, these initiatives have mostly remained localized or at best at the level of large institutions. A number of professional associations of doctors and other specialists’ group have given recommendations on adult vaccination;^16,17^ however, uptake has remained low and recommendations voluntary to be adopted by members of that professional association.

The lack of data, both on coverage of adult population with vaccines as well as on burden of VPDs in this population group is a major challenge. The limited data suggests that the adult vaccination coverage in country is negligible. Dash et al.^12^ have made suggestions such as formulation of national adult vaccination policy for India, which could be desirable yet not sufficient. A number of high-income countries have formal recommendations but coverage of adults with vaccines remains low; though relatively better coverage in high-risk population groups.^18^

All India Institute of Medical Sciences (AIIMS), Jodhpur, India (where one of the authors work, PB) had set up a dedicated Adult Vaccination center in January 2018. The data was analyzed to find that in period of first 12 months, a total of 1,388 persons attended Adult Vaccination clinic. Out of all, 583 (42%) were given TT Vaccines followed by 278 (20%) who received anti rabies vaccines. Both of these vaccines fall in the post-exposure category. For pre-exposure category, the vaccines utilized were yellow fever 208 (15%), hepatitis B 111 (8%), pneumococcal vaccine 97 (7%), typhoid 42 (3%) and influenza 14 (1%). The other vaccines were received by 56 (4%) people and included meningococcal vaccine, hepatitis A, varicella and OPV. This data from AIIMS, Jodhpur indicate that even with the availability of such services, the adults are not utilizing these services to the fullest. Similar was the experience when the Government of India offered Japanese Encephalitis (JE) vaccine to adult population and the turn up was only 50% of the adult population targeted.^11^ Clearly, there is a high need to improve the adult vaccination to reduce the burden of VPDs as well as cost of hospitalization and treatment.

It is expected that with ongoing efforts to improved coverage of vaccines for children, immunization coverage for the target age group is expected to reach 95% or beyond and can be sustained at that level. However, to reap the full benefit of available vaccines, additional populations including high-risk groups and adult need to be covered. There are a few possible next step. First, the NTAGI in India to provide recommendations for adult vaccination. Second, improved recording and reporting system for VPDs with age group wise data. Third, public as well as healthcare provider education and awareness on increased utilization of vaccines for adults. Fourth, systematic mechanisms for vaccination of high-risk adult population such as people with underlying chronic health conditions or those on long term treatment. Fifth, there is need for more operational research on understanding acceptance and practice toward adult vaccination in India. Sixth, education of healthcare providers at various levels and that of members of professional association in adult vaccination should be included in various training programs and curricula. Seventh, mainstream vaccination for all age groups into package of services offered through government primary healthcare facilities and that of health & wellness centers (HWCs) under Ayushman Bharat program of India.^19^

Immunization program coverage is often considered the best possible coverage with any health interventions. The global community has committed to achieve Universal Health Coverage (UHC) as part of Sustainable Development Goal (SDG) agenda 2030. The progress in India and globally on UHC is likely to be dependent upon how the benefit of proven and cost-effective interventions reaches to every person in need. Adult immunization as life course approach to health services would be a key to advance UHC in India & other low- and middle-income countries.

## References

[1] World Health Organization. The expanded program of Immunization. Geneva (Switzerland): WHO; 1974. https://www.who.int/immunization/programmes_systems/supply_chain/benefits_of_immunization/en/.

[2] Lahariya C. A brief history of vaccines & vaccination in India. Indian J Med Res. 2014;139:491–511. 24927336 PMC4078488

[3] International Vaccine Access Center (IVAC). Johns Hopkins Bloomberg School of Public Health; 2015 [accessed 2019 Oct 02]. http://www.jhsph.edu/research/centers-and-institutes/ivac/view-hub/IVAC-VIMS-Report-2015Dec.pdf

[4] Lahariya C. “Health system approach” for improving immunization program performance. J Family Med Prim Care. 2015;4:487–94. doi:10.4103/2249-4863.174263. 26985404 PMC4776597

[5] Govt of India. UIP webpage. [accessed 2019 Oct 02]. https://mohfw.gov.in/sites/default/files/5628564789562315.pdf

[6] Tricco AC, Zarin W, Cordoso R, Veronici A-A, Khan PA, Nincic V, Ghassemi M, Warren R, Sharpe JP, Page AV, et al. Efficacy, effectiveness, and safety of herpes zoster vaccines in adults aged 50 and older: systematic review and network meta-analysis. BMJ. 2018;363:k4782. doi:10.1136/bmj.k4029. 30361202 PMC6201212

[7] Govt of India. Universal immunization program: comprehensive multi-year strategic plan 2013-17. Nirman Bhawan (New Delhi): Ministry of Health & Family Welfare; 2013. https://www.who.int/immunization/programmes_systems/financing/countries/cmyp/india_cmyp_2013-17.pdf.

[8] Govt of India. Universal immunization program: comprehensive multi-year strategic plan 2018-22. Nirman Bhawan (New Delhi): Ministry of Health & Family Welfare; 2018. https://nhm.gov.in/New_Updates_2018/NHM_Components/Immunization/Guildelines_for_immunization/cMYP_2018-22_final_pdf.

[9] Lahariya C, Subramanya BP, Sosler S. An assessment of hepatitis B vaccine introduction in India: lessons for roll out and scale up of new vaccines in immunization programs. Indian J Public Health. 2013;57:8–14. doi:10.4103/0019-557X.111357. 23649136

[10] Gupta SK, Sosler S, Lahariya C. Introduction of Haemophilus influenzae type b (Hib) as pentavalent(DPT-HepB-Hib) vaccine in two states of India. Indian Pediatr. 2012;49:707–09. doi:10.1007/s13312-012-0151-0. 23024078

[11] Kumar P, Pisudde PM, Sarthi PP, Sharma MP, Keshri VR. Status and trend of acute encephalitis syndrome and Japanese encephalitis in Bihar, India. Natl Med J India. 2017;30:317–20. doi:10.4103/0970-258X.239070. 30117441

[12] Dash R, Agrawal A, Nagvekar V, Lele J, Pasquale AD, Kolhapure S Towards adult vaccination in India: a narrative literature review. [The article being published in this issue.] 10.1080/21645515.2019.1682842 PMC7227717 31746661

[13] Tiffay K, Jodar L, Kieny MP, Socquet M, LaForce FM. The evolution of the meningitis vaccine project. Clin Infect Dis. 2015;61(Suppl 5):S396–403. doi:10.1093/cid/civ594. 26553666 PMC4639496

[14] Govt of India. National vaccine policy 2011. Nirman Bhawan (New Delhi): Ministry of Health & Family Welfare; 4 2011.

[15] John TJ. India’s national technical advisory group on immunisation. Vaccine. 2010;28(Suppl 1):A88–90. doi:10.1016/j.vaccine.2010.02.041. 20413005

[16] Ramasubramanian V. Chapter 6. adult immunization in India. [accessed 2019 Oct 02]. http://www.apiindia.org/pdf/progress_in_medicine_2017/mu_06.pdf.

[17] Indian Society of Nephrology. ISN guidelines for vaccination in chronic kidney disease. Indian J Nephrol 2016;26(Suppl1):S1–S30.

[18] Centre for Disease Control and Prevention. Advisory committee on Vaccine preventable diseases. [accessed 2019 Oct 02]. https://www.cdc.gov/vaccines/imz-managers/coverage/adultvaxview/pubs-resources/NHIS-2016.html.

[19] Lahariya C. ‘Ayushman Bharat’ program and universal health coverage in India. Indian Pediatr. 2018;55:495–506. doi:10.1007/s13312-018-1341-1. 29978817

